# IL1RN genetic variations and risk of IPF: a meta-analysis and mRNA expression study

**DOI:** 10.1007/s00251-012-0604-6

**Published:** 2012-02-10

**Authors:** Nicoline M. Korthagen, Coline H. M. van Moorsel, Karin M. Kazemier, Henk J. T. Ruven, Jan C. Grutters

**Affiliations:** 1 Department of Pulmonology, St. Antonius Hospital, PO Box 2500, 3430 EM Nieuwegein, The Netherlands; 2 Division of Heart & Lungs, University Medical Centre Utrecht, Utrecht, The Netherlands; 3 Clinical Chemistry, St. Antonius Hospital, Nieuwegein, The Netherlands

**Keywords:** Interleukin-1, Interstitial lung disease, Meta-analysis, Single nucleotide polymorphism, mRNA expression

## Abstract

Idiopathic pulmonary fibrosis (IPF) is a rare and devastating lung disease of unknown aetiology. Genetic variations in the *IL1RN* gene, encoding the interleukin-1 receptor antagonist (IL-1Ra), have been associated with IPF susceptibility. Several studies investigated the variable number tandem repeat (VNTR) or single nucleotide polymorphisms rs408392, rs419598 and rs2637988, with variable results. The aim of this study was to elucidate the influence of polymorphisms in *IL1RN* on IPF susceptibility and mRNA expression. We performed a meta-analysis of the five case–control studies that investigated an *IL1RN* polymorphism in IPF in a Caucasian population. In addition, we investigated whether *IL1RN* mRNA expression was influenced by *IL1RN* polymorphisms. The VNTR, rs408392 and rs419598 were in tight linkage disequilibrium, with *D*′ > 0.99. Furthermore, rs2637988 was in linkage disequilibrium with the VNTR (*D*′ = 0.90). A haploblock of VNTR*2 and the minor alleles of rs408392and rs419598 was constructed. Meta-analysis revealed that this VNTR*2 haploblock is associated with IPF susceptibility both with an allelic model (odds ratio = 1.42, *p* = 0.002) and a carriership model (odds ratio = 1.60, *p* = 0.002). *IL1RN* mRNA expression was significantly influenced by rs2637988, with lower levels found in carriers of the (minor) GG genotype (*p* < 0.001). From this meta-analysis, we conclude that the VNTR*2 haploblock is associated with susceptibility to IPF. In addition, polymorphisms in *IL1RN* influence IL-1Ra mRNA expression, suggesting that lower levels of IL-1Ra predispose to developing IPF. Together these findings demonstrate that the cytokine IL-1Ra plays a role in IPF pathogenesis.

## Introduction

Idiopathic pulmonary fibrosis (IPF) is a rapidly progressing lung disease with unknown cause and a median survival of only 2.5 to 3.5 years (Gribbin et al. 2006; Mapel et al. 1998; Rudd et al. 2007). The disease is characterised by fibroblast growth, extracellular matrix deposition and remodelling of alveolar tissue, thereby disabling gas exchange across the alveolar epithelium (ATS/ERS 2002). IPF is a rare disease with a prevalence of 14 per 100,000 persons, but the incidence continues to rise and it is now an important cause of respiratory mortality (Navaratnam et al. 2011; Raghu et al. 2006). Familial and ethnic clustering support the theory that genetic variations influence IPF disease susceptibility, and identification of the genes involved can increase understanding of this complex disease (Grutters and du Bois 2005). Furthermore, because no effective treatment for IPF is available at present, genetic analysis may also reveal a novel target for therapy.

Interleukin (IL)-1 is a proinflammatory and profibrotic cytokine that exists in two forms: IL-1α and IL-1β. Interleukin-1 receptor antagonist (IL-1Ra) is an inhibitor IL-1 by competitive binding to the IL-1 receptor. The *IL1RN* gene, coding for IL-1Ra protein, has been implicated in IPF susceptibility (Whyte et al. 2000). Genetic variations in *IL1RN*, *IL1A* and *IL1B* have been associated with ulcerative colitis, gastric cancer and rheumatoid arthritis (Lee et al. 2009; Peleteiro et al. 2010; Queiroz et al. 2009). Variations in these genes can modulate the effectiveness of IL-1 signalling and thereby predispose to disease.

Several polymorphisms in *IL1RN* have been investigated in IPF case–control studies. Whyte et al. showed a significant association between IPF susceptibility and the single nucleotide polymorphism (SNP) rs419598 (also known as +2018) in two populations (Whyte et al. 2000). Others investigated a variable number tandem repeat (VNTR), rs408392 or rs2637988 and found that the genotype distributions of the VNTR and rs408392 were not significantly different between patients and controls (Barlo et al. 2011; Hutyrova et al. 2002; Riha et al. 2004). However, in the largest of these studies, a trend was observed with rs408392, and rs2637988 was found to be significantly associated with IPF susceptibility (Barlo et al. 2011). In a rare disease like IPF, validation of genetic associations is hampered by the small sample size of available cohorts. To establish whether IL1RN is associated with the risk of IPF, we performed a meta-analysis of the five case–control studies. Although these five studies investigated different polymorphisms, they could be combined in a meta-analysis because the polymorphisms are in tight linkage disequilibrium.

At present, it is unclear how polymorphisms in *IL1RN* predispose to disease. To compare the functional effects before disease onset, we determined genotype-dependent mRNA expression for the VNTR and rs2637988 in healthy controls.

## Material and methods

### Study selection

For this meta-analysis, we included all five case–control studies that investigated the association between polymorphisms in *IL1RN* and IPF in Caucasian populations (Table 1). In the English population, rs419598 (chromosome position 113887207) was determined in 88 patients and 88 controls (Whyte et al. 2000). The same SNP, rs419598, was determined in the Italian population in 61 patients and 103 controls (Whyte et al. 2000). In the Czech population, the VNTR (chromosome position 113888106) was determined in 54 patients and 199 controls (Hutyrova et al. 2002). The VNTR was also determined in the Australian population in 22 patients and 140 controls, but only allele frequencies were given and no individual genotypes were available (Riha et al. 2004). In the Dutch population, rs408392 (chromosome position 113887458) and rs2637988 (chromosome position 113876779) were determined in 77 patients and 349 controls (Barlo et al. 2011). In total, allele frequencies were available for 302 patients with IPF and 879 controls. Individual genotypes were available for 280 patients and 739 controls.

**Table 1:** Characteristics of the association studies on *IL1RN* and IPF

Study	Origin of cohort	Patients (*n*)	Controls (*n*)	Polymorphism	RAF patients	RAF controls	*p* value (allelic association)
Whyte et al. (2000a)	Britain	88	88	rs419598	0.28	0.17	0.02
Whyte et al. (2000b)	Italy	61	103	rs419598	0.33	0.20	0.01
Hutyrova et al. (2002)	Czech Rep.	54	199	VNTR	0.31	0.30	0.90
Riha et al. (2004)	Australia	22	140	VNTR	0.23	0.25	0.77
Barlo et al. (2011)	The Netherlands	77	349	rs408392	0.32	0.26	0.09
				rs2637988	0.47	0.38	0.04

*RAF* risk allele frequency, *NA* not available

The VNTR (rs2234663) and rs408392 are located in intron 2 (identical to intron 3 in the extended gene encoding the intracellular isoform of IL-1Ra (Raitala et al. 2006)); rs419598 is located at position +2018 in exon 2. rs2637988 is located upstream of *IL1RN* near several transcription factor binding sites.

The studies were identified using a PUBMED search using the terms ‘polymorphism’, ‘Genetic association’, ‘pulmonary fibrosis’ and ‘fibrosing alveolitis’. Only studies on idiopathic pulmonary fibrosis (also called cryptogenic fibrosing alveolitis) were included. One study was not a case–control study but only reported associations with disease phenotype and was excluded (Vasakova et al. 2007).

The populations included in this study were tested for allelic association with disease (Table 1) using Pearson’s goodness-of-fit chi-square as implemented online at http://ihg2.helmholtz-muenchen.de/cgi-bin/hw/hwa1.pl. This could not be done for the Australian population because individual genotypes were not available, and therefore, we included the *p* value as reported in their study in Table 1.

### Measurement of linkage disequilibrium

We investigated the linkage disequilibrium between the VNTR, rs419598, rs408392 and rs2637988. Because there is no HapMap data available for the VNTR, we compared the genotypes for the VNTR, rs408392 and rs2637988 in the Dutch cohort of 349 healthy controls (Table 1). Genotypes for rs408392 and rs2637988 were retrieved from the study by Barlo et al. (2011). The VNTR polymorphism was determined by polymerase chain reaction (PCR). Primers for the VNTR region were sense 5′-ACTCATGGCCTTGTTCATT and antisense 5′-AAAACTAAAATCCCGAGGTC (Sigma-Aldrich, St. Louis, MO, USA). PCR products were run on a 1.5% agarose gel. A 25- and a 200-base pair ladder were used to discern the number of 86-base pair repeats. The VNTR includes a variable number of repeats. The VNTR allele 2 (VNTR*2) is the shortest with only two repeats. The other alleles correspond to between three and six repeats.

Because rs419598 was not determined in the Dutch population, genotypes for the European HapMap population were retrieved for rs408392 and rs419598. We determined the linkage disequilibrium using the computer program Haploview 4.1 (Broad Institute of MIT and Harvard, USA).

### Meta-analysis

For the meta-analysis, we combined the case–control data from the VNTR, rs408392 and rs419598 (+2018) in *IL1RN* (Table 2) from five populations. The case and control populations were tested for violation of Hardy–Weinberg equilibrium.

**Table 2:** Data used in the meta-analysis

		Allele frequency model		Dominant model	
		Risk alleles/non-risk alleles (*n*)		Risk carriers/non-risk carriers (*n*)	
Study	Origin of cohort	Patients	Controls	Patients	Controls
Whyte et al. (2000a)	Britain	49/127	30/146	39/49	28/60
Whyte et al. (2000b)	Italy	40/82	42/164	35/26	37/66
Hutyrova et al. (2002)	Czech Rep.	33/75	119/279	27/27	96/103
Riha et al. (2004)	Australia	10/34	70/210	NA	NA
Barlo et al. (2011)	The Netherlands	50/104	180/518	43/34	155/194

Alleles refers to the number of alleles in the population; carriers refers to the number of individuals carrying the risk allele

*NA* not available

The meta-analysis was performed using R software (version 2.9.2, Catmap package, version 1.6, www.r-project.org). Both a random effects (DerSimonian and Laird method) and a fixed effects analysis (Mantel–Haenszel method) of the case–control data were performed. To test for heterogeneity, the Cochrane Q test and I^2^ was used. In addition, a leave-one-out sensitivity analysis was performed. When all five populations were combined, we could only use an allele-based model because individual genotypes were not available for the Australian population. To investigate whether the risk allele has a dominant or recessive influence on disease development in the population, both models were tested. Due to the absence of individual genotypes, the Australian population was removed and the remaining number of individuals carrying one or more risk alleles was used in the analysis.

### RNA expression

We used thawed peripheral blood mononuclear cells (PBMC) from 38 healthy controls (23 males and 15 females, mean age 22.5 years). The expression of *IL1RN* mRNA was analysed by quantitative RT-PCR amplification as described previously (Heron et al. 2009). Briefly, total RNA was isolated using an RNeasy microkit (Qiagen, Venlo, The Netherlands) and cDNA was made using the I-script cDNA synthesis kit (Bio-Rad, Veenendaal, The Netherlands). Primers used for expression analysis of *IL1RN* were forward 5′-GAAGATGTGCCTGTCCTGTGTC and reverse 5′-CGCTTGTCCTGCTTTCTGTTC (Sigma-Aldrich). The copy numbers were normalised by the housekeeping gene β-actin (forward 5′-AGCCTCGCCTTTGCCGA and reverse 5′-CTGGTGCCTGGGGCG). SPSS and GraphPad Prism software were used to test whether RNA expression differed per genotype. A difference with a *p* < 0.05 was considered statistically significant.

## Results

### Measurement of linkage disequilibrium

**Fig. 1:**
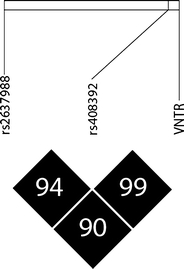
Linkage plot for polymorphisms in *IL1RN* showing *D*′

Analysis of European HapMap population genotypes showed that the two biallelic SNPs rs408392 and rs419598 are in complete linkage disequilibrium (*D*′ = 1). There was tight linkage disequilibrium between the VNTR and rs408392 in our Dutch control population (*D*′ = 0.99, Fig. 1). We observed in the Dutch cohort, that in all individuals, the longer VNTR alleles with three or more repeats corresponded completely to the major allele of rs408392 and of rs419598, while the VNTR*2 allele corresponded to the minor alleles of these SNPs.

Therefore, the VNTR, rs408392 and rs419598 are part of a haploblock, and for analysis, we combined the linked alleles: we named the combination of risk alleles ‘VNTR*2 haploblock’ and this contained the VNTR*2 and the minor alleles of rs419598 and rs408392.

Linkage disequilibrium between rs2637988 and the VNTR (*D*′ = 0.90) and between rs2637988 and rs408392 (*D*′ = 0.94) was high (Fig. 1), but the difference in frequencies between the coupled alleles was substantial, and therefore, rs2637988 was not included in the meta-analysis.

### Meta-analysis

The data used for the meta-analysis are shown in Table 2. Cases and controls were individuals of European ancestry from Britain, Italy, Czech Republic, Australia and The Netherlands. The genotypes from all populations were in Hardy–Weinberg equilibrium. Pooling of the populations resulted in a risk allele frequency of the VNTR*2 haploblock of 0.30 in patients (*n* = 302) and 0.25 in controls (*n* = 879).

**Fig. 2:**
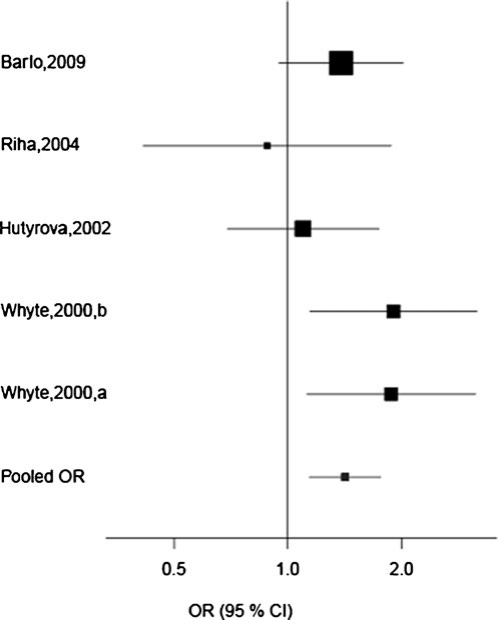
Fixed effects meta-analysis with an allele frequency model of the effect of *IL1RN* on IPF susceptibility. Individual study odds ratios (*ORs*) are shown as well as the pooled OR for the VNTR*2 haploblock (VNTR, rs408392, rs419598). The pooled OR was 1.42 (CI 1.14–1.76, *p* = 0.002)

A significant association between the VNTR*2 haploblock and IPF was observed using an allele frequency model with a fixed effects meta-analysis. The pooled odds ratio (OR) was 1.42 (95% confidence interval (CI) 1.14–1.76, *p* = 0.002, *χ* ^2^ = 9.93) (Fig. 2). Sensitivity analysis showed that the association remained significant after sequential removal of each one of the studies. Cochrane Q test indicated there was no significant variability/heterogeneity among the studies in this meta-analysis (*χ* ^2^ = 5.2, *p* = 0.27). The I^2^ was 23%, indicating that inconsistency across studies was low. Although no heterogeneity was seen across studies, the random effect models were run and ORs and CIs were almost identical to those seen in the fixed effects analysis.

Under the dominant genetic model, comparing non-carriers to carriers of the VNTR*2 haploblock, the pooled OR was 1.60 (95% CI 1.20–2.14, *p* = 0.002, *χ* ^2^ = 10.01) under a fixed effects assumption. The relative risk to develop IPF for an individual carrying the VNTR*2 haploblock was 1.29 (95% CI 1.05–1.57, *p* = 0.01) based on the four populations in this analysis. The recessive genetic model compares carriers of the VNTR*1 haploblock with homozygous carriers of VNTR*2 haploblock and did not result in a significant association.

### mRNA expression analysis

**Fig. 3:**
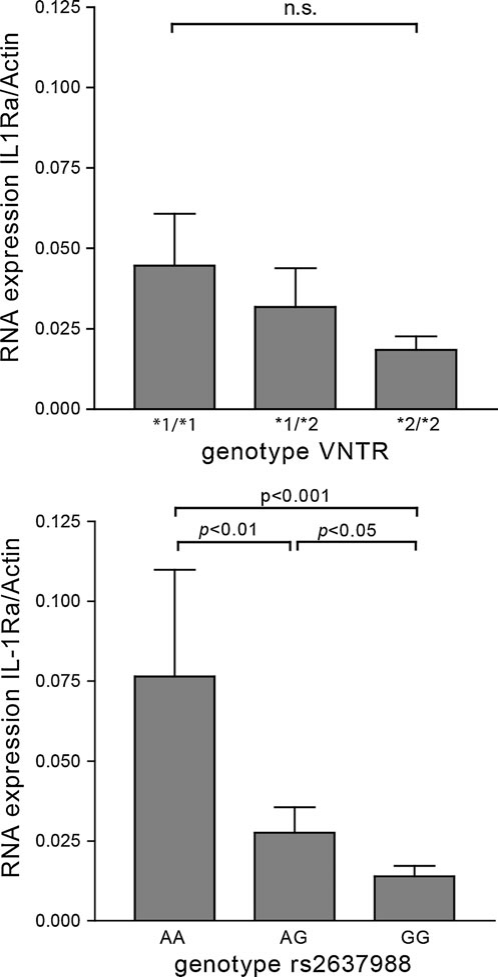
The effect of *IL1RN* genotype on mRNA expression. IL-1Ra mRNA expression in PBMC from healthy controls normalised to actin expression. There was no significant effect of *IL1RN* VNTR genotype on mRNA expression. A significant association between rs2637988 and mRNA expression was found. *Error bars* represent standard error of the mean. VNTR*1 corresponds to four repeats and VNTR*2 corresponds to two repeats of the VNTR polymorphism

We analysed IL-1Ra mRNA expression in relation to *IL1RN* genotypes. A dosage effect tendency of the VNTR polymorphism on *IL1RN* mRNA expression is shown in Fig. 3, but this did not reach significance (*p* = 0.30). Rs2637988 A/G was significantly associated with a difference in IL-1Ra mRNA expression levels in healthy PBMC (Fig. 3). Significantly lower mRNA expression levels were found in control subjects with the minor GG genotype compared to controls with the AG genotype (*p* < 0.05) or the AA genotype (*p* < 0.001).

## Discussion

This meta-analysis showed that variations in *IL1RN* are significantly associated with an increased risk of developing IPF. In our linkage analysis, we found that there was tight linkage disequilibrium between the VNTR, rs408392 and rs419598, allowing the polymorphisms to be combined into a VNTR*2 haploblock for the meta-analysis of five IPF populations. The pooled odds ratio of the allele frequency model showed that the VNTR*2 haploblock was significantly associated with IPF. However, in the Italian and Dutch populations, the largest association was reported for carriership of the minor allele, corresponding to the VNTR*2 haploblock (Barlo et al. 2011; Whyte et al. 2000). With a dominant genetic model, the meta-analysis showed an even stronger association with IPF than with the allelic model (OR = 1.60 vs. OR = 1.42). This confirms that carriership of the risk allele was most associated with IPF. Individuals that are homo- or heterozygous for the risk allele are at an increased risk of developing IPF. Calculation of the relative risk showed that carriership of the risk allele confers a 29% increase in the risk of developing IPF. Only individuals without a risk allele, the majority of the population, are protected from developing IPF.

The effects of IL-1 are mediated by two protein isoforms, IL-1α and IL-1β, who act through binding to the IL-1 receptor. Their effects are counterbalanced by the IL-1Ra protein that binds to the receptor but does not transduce any signal. The IL-1 effector response is determined by the balance between these proteins (Barksby et al. 2007; Ludwiczek et al. 2004; Sekiyama et al. 1994). The ratio between IL1-Ra and IL-1 was found to be lower in serum from IPF patients and in macrophages from IPF lungs (Barlo et al. 2011; Mikuniya et al. 1997). Previous studies have found that the IL-1Ra/IL-1 ratio is influenced by genetic variations in *IL1RN* (Barlo et al. 2011; Carter et al. 2004). Higher IL-1 protein levels were found in gastric mucosa from *Helicobacter pylori*-infected individuals that carried VNTR*2 (Garcia-Gonzalez et al. 2009), while lower IL-Ra protein levels were found in biopsies from ulcerative colitis patients with VNTR*2 (Carter et al. 2004). Together, this indicates that carriers of VNTR*2 have low levels of IL-1Ra but higher IL-1 levels. The VNTR is a variation in an 86-base pair repeat in an intron of the *IL1RN* gene. The change in mRNA length caused by the VNTR could have an effect on its processing and stability. Analysis of *IL1RN* mRNA expression in healthy controls showed that there was a suggestive effect of the VNTR polymorphism (and thus rs408392 and rs419598), but this did not reach significance. The dosage effect observed for the VNTR might be caused by linkage to rs2637988 because rs2637988 is in nearly complete linkage disequilibrium with the VNTR. We found that rs2637988 GG was significantly associated with lower *IL1RN* mRNA expression. In addition, in the study by Barlo et al., the rs2637988 G allele was significantly associated with IPF susceptibility (OR = 1.95) whereas rs408392 showed only a trend towards significance (Barlo et al. 2011). Together, this indicates that the *IL1RN* rs2637988 polymorphism might have a greater effect on IPF disease development than the VNTR*2 haploblock. The study by Barlo et al. (2011) also showed that rs2637988 G was associated with a lower ratio of IL-1Ra to IL-1β. Enhanced expression of IL-1β has been found in alveolar macrophages and pneumocytes in patients with acute pulmonary fibrotic diseases (Pan et al. 1996). In animal models of IPF, IL-1β levels are elevated in mice with bleomycin-induced lung fibrosis (Gasse et al. 2007; Hoshino et al. 2009). Blocking the IL-1 receptor with IL-1Ra reduced bleomycin-induced inflammation and prevented fibrosis in these mice (Gasse et al. 2007; Piguet et al. 1993). Transient overexpression of IL-1β caused acute lung injury resulting in pulmonary fibrosis in rats (Kolb et al. 2001). Thus, relatively low levels of IL-1Ra would fail to prevent the pro-fibrotic functions of IL-1, and this could play an important role in IPF disease aetiology. In light of these findings, treatment of IPF patients with an IL-1 antagonist, like anakinra, should be considered. So far, treatment with IL-1 blocking agents has been safe and effective in rheumatoid arthritis, although the increased risk of respiratory infection may mean caution is called for (Geyer and Muller-Ladner 2010). In addition, it is possible that genetic variations in the IL1A and IL1B genes also influence the balance between IL-1 and IL-1Ra and could therefore play a role in IPF susceptibility. Future studies are needed to evaluate the role of polymorphisms in these genes and their interaction with each other.

In conclusion, our meta-analysis shows that polymorphisms associated with the *IL1RN* VNTR increase susceptibility to IPF. The IL1RN risk allele is associated with lower levels of IL-1Ra. After the recent association of the MUC5B gene with IPF (Seibold et al. 2011), this is the second largest IPF association study. The role of IL-1Ra in preventing fibrosis supports the notion that insufficiently expressed IL-1Ra can permit fibrogenesis to occur, thereby predisposing to IPF.

## References

[CR1] ATS/ERS (2002). American Thoracic Society/European Respiratory Society International Multidisciplinary Consensus Classification of the idiopathic interstitial pneumonias. This joint statement of the American Thoracic Society (ATS), and the European Respiratory Society (ERS) was adopted by the ATS board of directors, June 2001 and by the ERS Executive Committee, June 2001. Am J Respir Crit Care Med.

[CR2] Barksby HE, Lea SR, Preshaw PM, Taylor JJ (2007). The expanding family of interleukin-1 cytokines and their role in destructive inflammatory disorders. Clin Exp Immunol.

[CR3] Barlo NP, van Moorsel CH, Korthagen NM, Heron M, Rijkers GT, Ruven HJ, van den Bosch JM, Grutters JC (2011). Genetic variability in the IL1RN gene and the balance between interleukin (IL)-1 receptor agonist and IL-1beta in idiopathic pulmonary fibrosis. Clin Exp Immunol.

[CR4] Carter MJ, Jones S, Camp NJ, Cox A, Mee J, Warren B, Duff GW, Lobo AJ, di Giovine FS (2004). Functional correlates of the interleukin-1 receptor antagonist gene polymorphism in the colonic mucosa in ulcerative colitis. Genes Immun.

[CR5] Garcia-Gonzalez MA, Aisa MA, Strunk M, Benito R, Piazuelo E, Jimenez P, Sopena F, Lanas A (2009). Relevance of IL-1 and TNF gene polymorphisms on interleukin-1beta and tumor necrosis factor-alpha gastric mucosal production. Hum Immunol.

[CR6] Gasse P, Mary C, Guenon I, Noulin N, Charron S, Schnyder-Candrian S, Schnyder B, Akira S, Quesniaux VF, Lagente V, Ryffel B, Couillin I (2007). IL-1R1/MyD88 signaling and the inflammasome are essential in pulmonary inflammation and fibrosis in mice. J Clin Invest.

[CR7] Geyer M, Muller-Ladner U (2010). Actual status of antiinterleukin-1 therapies in rheumatic diseases. Curr Opin Rheumatol.

[CR8] Gribbin J, Hubbard RB, Le JI, Smith CJ, West J, Tata LJ (2006). Incidence and mortality of idiopathic pulmonary fibrosis and sarcoidosis in the UK. Thorax.

[CR9] Grutters JC, du Bois RM (2005). Genetics of fibrosing lung diseases. Eur Respir J.

[CR10] Heron M, Grutters JC, van Moorsel CH, Ruven HJ, Kazemier KM, Claessen AM, van den Bosch JM (2009). Effect of variation in ITGAE on risk of sarcoidosis, CD103 expression, and chest radiography. Clin Immunol.

[CR11] Hoshino T, Okamoto M, Sakazaki Y, Kato S, Young HA, Aizawa H (2009). Role of proinflammatory cytokines IL-18 and IL-1beta in bleomycin-induced lung injury in humans and mice. Am J Respir Cell Mol Biol.

[CR12] Hutyrova B, Pantelidis P, Drabek J, Zurkova M, Kolek V, Lenhart K, Welsh KI, du Bois RM, Petrek M (2002). Interleukin-1 gene cluster polymorphisms in sarcoidosis and idiopathic pulmonary fibrosis. Am J Respir Crit Care Med.

[CR13] Kolb M, Margetts PJ, Anthony DC, Pitossi F, Gauldie J (2001). Transient expression of IL-1beta induces acute lung injury and chronic repair leading to pulmonary fibrosis. J Clin Invest.

[CR14] Lee YH, Ji JD, Song GG (2009). Association between interleukin 1 polymorphisms and rheumatoid arthritis susceptibility: a metaanalysis. J Rheumatol.

[CR15] Ludwiczek O, Vannier E, Borggraefe I, Kaser A, Siegmund B, Dinarello CA, Tilg H (2004). Imbalance between interleukin-1 agonists and antagonists: relationship to severity of inflammatory bowel disease. Clin Exp Immunol.

[CR16] Mapel DW, Hunt WC, Utton R, Baumgartner KB, Samet JM, Coultas DB (1998). Idiopathic pulmonary fibrosis: survival in population based and hospital based cohorts. Thorax.

[CR17] Mikuniya T, Nagai S, Shimoji T, Takeuchi M, Morita K, Mio T, Satake N, Izumi T (1997). Quantitative evaluation of the IL-1 beta and IL-1 receptor antagonist obtained from BALF macrophages in patients with interstitial lung diseases. Sarcoidosis Vasc Diffuse Lung Dis.

[CR18] Navaratnam V, Fleming KM, West J, Smith CJ, Jenkins RG, Fogarty A, Hubbard RB (2011). The rising incidence of idiopathic pulmonary fibrosis in the U.K. Thorax.

[CR19] Pan LH, Ohtani H, Yamauchi K, Nagura H (1996). Co-expression of TNF alpha and IL-1 beta in human acute pulmonary fibrotic diseases: an immunohistochemical analysis. Pathol Int.

[CR20] Peleteiro B, Lunet N, Carrilho C, Duraes C, Machado JC, La VC, Barros H (2010). Association between cytokine gene polymorphisms and gastric precancerous lesions: systematic review and meta-analysis. Cancer Epidemiol Biomarkers Prev.

[CR21] Piguet PF, Vesin C, Grau GE, Thompson RC (1993). Interleukin 1 receptor antagonist (IL-1ra) prevents or cures pulmonary fibrosis elicited in mice by bleomycin or silica. Cytokine.

[CR22] Queiroz DM, Oliveira AG, Saraiva IE, Rocha GA, Rocha AM, das Gracas Pimenta SM, Guerra JB, Dani R, Ferrari ML, Castro LP (2009). Immune response and gene polymorphism profiles in Crohn's disease and ulcerative colitis. Inflamm Bowel Dis.

[CR23] Raghu G, Weycker D, Edelsberg J, Bradford WZ, Oster G (2006). Incidence and prevalence of idiopathic pulmonary fibrosis. Am J Respir Crit Care Med.

[CR24] Raitala A, Hurme M, Pessi T, Eklund C, Vandenbroeck K (2006). The IL1 cluster. Cytokine gene polymorphisms in multifactorial conditions.

[CR25] Riha RL, Yang IA, Rabnott GC, Tunnicliffe AM, Fong KM, Zimmerman PV (2004). Cytokine gene polymorphisms in idiopathic pulmonary fibrosis. Intern Med J.

[CR26] Rudd RM, Prescott RJ, Chalmers JC, Johnston ID (2007). British Thoracic Society Study on cryptogenic fibrosing alveolitis: response to treatment and survival. Thorax.

[CR27] Seibold MA, Wise AL, Speer MC, Steele MP, Brown KK, Loyd JE, Fingerlin TE, Zhang W, Gudmundsson G, Groshong SD, Evans CM, Garantziotis S, Adler KB, Dickey BF, du Bois RM, Yang IV, Herron A, Kervitsky D, Talbert JL, Markin C, Park J, Crews AL, Slifer SH, Auerbach S, Roy MG, Lin J, Hennessy CE, Schwarz MI, Schwartz DA (2011). A common MUC5B promoter polymorphism and pulmonary fibrosis. N Engl J Med.

[CR28] Sekiyama KD, Yoshiba M, Thomson AW (1994). Circulating proinflammatory cytokines (IL-1 beta, TNF-alpha, and IL-6) and IL-1 receptor antagonist (IL-1Ra) in fulminant hepatic failure and acute hepatitis. Clin Exp Immunol.

[CR29] Vasakova M, Striz I, Dutka J, Slavcev A, Jandova S, Kolesar L, Sulc J (2007). Cytokine gene polymorphisms and high-resolution-computed tomography score in idiopathic pulmonary fibrosis. Respir Med.

[CR30] Whyte M, Hubbard R, Meliconi R, Whidborne M, Eaton V, Bingle C, Timms J, Duff G, Facchini A, Pacilli A, Fabbri M, Hall I, Britton J, Johnston I, Di GF (2000). Increased risk of fibrosing alveolitis associated with interleukin-1 receptor antagonist and tumor necrosis factor-alpha gene polymorphisms. Am J Respir Crit Care Med.

